# Real-world impact of integrating HIV assisted partner services into 31 facilities in Kenya: a single-arm, hybrid type 2 implementation-effectiveness study

**DOI:** 10.1016/S2214-109X(23)00153-5

**Published:** 2023-05

**Authors:** Monisha Sharma, Brienna Naughton, Harison Lagat, George Otieno, David A Katz, Beatrice M Wamuti, Sarah Masyuko, Rose Bosire, Mary Mugambi, Unmesha Roy Paladhi, Bryan J Weiner, Edward Kariithi, Carey Farquhar

**Affiliations:** Department of Global Health, University of Washington, Seattle, WA, USA; Department of Global Health, University of Washington, Seattle, WA, USA; PATH Kenya, Kisumu, Kenya; PATH Kenya, Kisumu, Kenya; Department of Global Health, University of Washington, Seattle, WA, USA; Harvard T H Chan School of Public Health, Boston, MA, USA; Department of Global Health, University of Washington, Seattle, WA, USA; Kenya Ministry of Health, Nairobi, Kenya; Kenya Medical Research Institute, Nairobi, Kenya; Kenya Ministry of Health, Nairobi, Kenya; Department of Global Health, Department of Epidemiology, University of Washington, Seattle, WA, USA; Department of Global Health, University of Washington, Seattle, WA, USA; PATH Kenya, Kisumu, Kenya; Department of Global Health, Department of Epidemiology, Department of Medicine, University of Washington, Seattle, WA, USA

## Abstract

**Background:**

Assisted partner services (APS), or exposure notification and HIV testing for sexual partners of individuals diagnosed with HIV (index clients), have been shown to be safe and effective in clinical trials. We assessed the real-world effectiveness of APS when integrated into HIV clinics in western Kenya.

**Methods:**

In this single-arm, hybrid type 2 implementation science study, we facilitated APS implementation in 31 health facilities in Kenya by training existing health-care staff. We focused on male partner outcomes to assess the impact of APS in reaching male individuals in sub-Saharan Africa, who have lower rates of HIV testing than female individuals. Female individuals (aged ≥18 years or emancipated minor) who tested positive for HIV at participating facilities in Kenya were offered APS; consenting female participants provided contact information for all male sexual partners in the past 3 years. Male partners were notified of their potential HIV exposure and offered a choice of community-based or facility-based HIV testing services (HTS). Female index clients and male partners with HIV were followed up at 6 weeks, 6 months, and 12 months after enrolment, to assess linkage to antiretroviral treatment. Viral load was assessed at 12 months.

**Findings:**

Between May 1, 2018, and March 31, 2020, 32 722 female individuals received HTS; 1910 (6%) tested positive for HIV, of whom 1724 (90%) received APS. Female index clients named 5137 male partners (median 3 per index [IQR 2–4]), of whom 4422 (86%) were reached with exposure notification and HTS. 524 (12%) of the male partners tested were newly diagnosed with HIV and 1292 (29%) reported a previous HIV diagnosis. At 12 months follow-up, 1512 (88%) female index clients and 1621 (89%) male partners with HIV were taking ART, with few adverse events: 25 (2%) female index clients and seven (<1%) male partners reported intimate partner violence, and 60 (3%) female index clients and ten (<1%) male partners reported relationship dissolution.

**Interpretation:**

Evidence from this real-world APS scale-up project shows that APS is a safe, acceptable, and effective strategy to identify males with HIV and retain them in care.

**Funding:**

The US National Institutes of Health.

## Introduction

HIV continues to be a substantial cause of morbidity and mortality, and sub-Saharan Africa accounts for more than 70% of new HIV infections globally. Despite scale-up of widespread HIV testing, an estimated 10–33% of individuals with HIV in sub-Saharan Africa are unaware of their HIV status.^1,2^ In particular, male individuals in sub-Saharan Africa are less likely to test for HIV than female individuals, resulting in delayed linkage to antiretroviral therapy (ART), poorer clinical outcomes, and increased likelihood of onward transmission.^3^ Strategies to reach undiagnosed male individuals with HIV testing services (HTS) are urgently needed to meet the UNAIDS 95–95–95 goals by 2030.^4^ Individuals with HIV are generally identified through facility-based HIV testing; however, testing coverage has been insufficient to reduce the epidemic, particularly in male individuals, a WHO priority population.^5,6^

Assisted partner services (APS), or notification and HIV testing for sexual partners of individuals diagnosed with HIV (index clients), is a promising targeted strategy to increase awareness of HIV status. Types of APS include provider notification, in which health-care providers contact sexual partners of consenting index clients and offer testing; contract referral, in which index clients are given a set amount of time to notify partners, after which health-care providers conduct notification; and dual referral, in which index clients and health-care providers coordinate to contact partners. APS is often implemented as a combination of these options. Clinical trials and demonstration projects in sub-Saharan Africa show that APS is a safe, efficient, and effective strategy to reach individuals who have been exposed to HIV with testing and linkage to care.^7-12^ A meta-analysis^13^ reported that APS was associated with a 50% increase in HIV testing among partners compared with standard of care, with few adverse events. In Kenya, a cluster-randomised clinical trial^9^ reported that APS provision resulted in higher partner HIV testing, identification of newly diagnosed HIV in partners, linkage to care, and first-time testers compared with passive referral. APS could be a particularly effective strategy to reach male individuals who might otherwise not access timely HIV testing. In 2016, WHO recommended offering APS as part of routine HIV services.^14^ Evidence from real-world APS programmes, particularly on long-term outcomes, is needed to inform programmatic decision making.

In this single-arm, hybrid type 2 implementation-effectiveness study, we assessed the real-world performance of APS when integrated into government-operated facilities in Kenya. We describe demographics, APS uptake, ART linkage and retention, and viral suppression among female index clients and their male partners. We focus on male partner outcomes to assess the impact of APS in reaching male individuals with HIV testing and care. This study yields evidence of real-world implementation of integrated APS in sub-Saharan Africa and is among the first to provide long-term data on ART retention and viral suppression.

## Methods

### Study design and participants

This APS scale-up project was a single-arm, hybrid type 2 implementation science study^15^ conducted in western Kenya, a region with high HIV prevalence (15%).^16^ The implementation team consisted of collaborators from the University of Washington (Seattle, WA, USA), PATH Kenya (a non-governmental organisation), and the Ministry of Health of Kenya; details of the implementation partners are specified in the appendix (p 2). The study protocol has been previously published.^17^ We implemented APS for the first time in 31 health facilities: 16 in Kisumu county, from May 1, 2018, and 15 in Homa Bay county, from Nov 1, 2018, with a combination of low-volume and high-volume facilities ranging from primary care to tertiary care. Female individuals were eligible for inclusion if they were aged 18 years or older (or emancipated minors aged ≥15 years), not at risk of intimate partner violence (assessed via questionnaire; appendix p 3), not pregnant, newly diagnosed with HIV, not receiving ART, and reported at least one sexual partner within the past 3 years. Male partners were eligible for testing through APS if they were aged 18 years or older (or emancipated minors aged ≥15 years). The present analysis includes 12 months of follow-up data from female index clients enrolled in APS from the start of the programme until March 31, 2020, and male partners elicited from these female index clients.

The study was approved by the Kenyatta National Hospital Ethics and Research Committee (P465/052017), the University of Washington Institutional Review Board (STUDY00002420), and the PATH Institutional Review Board. All participants provided informed consent.

At the start of the project, our implementation team held working group meetings with in-country stakeholders and experts to develop guidelines for integrating APS within HIV clinics in Kenya. These discussions were informed by the 2016 WHO guidelines on partner notification,^18^ as well as strategies used in an APS cluster-randomised controlled trial in Kenya,^9^ and later formed the basis of the Kenya Ministry of Health APS guidelines. To provide training, mentorship, and ongoing quality assurance, we used eight health advisors who had delivered APS in the study by Cherutich and colleagues.^9^ The health advisers were employed by PATH (implementing partner) and led a 3-day training for HTS counsellors (government health-care workers) and their managers from participating clinics. Topics included APS eligibility screening, intimate partner violence assessment, counselling, partner elicitation strategies, best practices for engaging with partners to provide exposure notification, and partner tracing. Health advisors subsequently provided intensive one-on-one training to HTS counsellors at each clinic, supporting the entire APS process, including counselling, elicitation, and tracing. HTS counsellors conducted APS procedures jointly with health advisors before conducting the process on their own. Health advisors provided ongoing support and mentorship for APS for the first 6–8 months of implementation and then slowly reduced their support. HTS managers (trained by the health advisors) then began taking over mentorship. Additionally, senior HTS counsellors (ie, those who had already received training for APS) took over training and mentorship for newly hired HTS counsellors. The Ministry of Health of Kenya also began training and certifying APS trainers of trainers, to support HTS counsellors and ensure APS was conducted according to Kenya Ministry of Health guidelines. For the first 2 years of APS implementation, we employed eight health advisors to support participating clinics; we reduced the number to four health advisors in 2020, and then to two in 2022. We continued collecting data for the APS project until 2022; however, the current paper reports pre-COVID-19 data only. We also provided HTS counsellors with several ongoing trainings on topics, including partner elicitation skills and engagement of partners, which led to improvements in numbers of partners elicited and enrolled during the study. Details of APS training, mentorship, as well as monitoring and evaluation in Kenya have been previously published.^19^

We focused on strengthening systems and capacity building, to enhance the likelihood of APS programme success. Although we did not provide performance incentives, we funded salaries of HTS counsellors and health advisors, initial and ongoing trainings, and telephone and in-person partner tracing and notification. This funding was delivered via PATH, our implementing partner mandated by US President’s Emergency Plan for AIDS Relief (also known as PEPFAR) to deliver HIV services. The approach of using a non-governmental organisation to implement a new intervention on behalf of a donor and the government is routinely applied in Kenya to support national and county scale-up of health services. This strategy improves existing health system capacity to deliver interventions instead of creating parallel systems.

### Procedures and outcomes

Briefly, our objective was to evaluate APS when integrated into health facilities in Kenya using government HTS counsellors who perform APS alongside their routine duties. Although APS was offered to both male individuals and female individuals diagnosed with HIV (index clients), we only collected programme data for female index clients and their male partners. We also collected data on female partners of male partners, which are not reported in the present study. HTS counsellors offered APS to eligible individuals at the time of HIV diagnosis. Consenting female participants were asked to provide names and contact information for all partners in the past 3 years. Full details are specified in the protocol.^17^

HTS counsellors contacted male partners via telephone or in-person to notify them of their potential exposure and offer HIV testing. Partners were told that they might have been exposed to HIV but were not given specific information regarding their exposure (ie, when or where they might have been exposed to HIV) nor identifying information about the index client. Male partners who tested positive for HIV were also offered APS and asked to provide contact information for their female partners. HTS counsellors provided exposure notification and HIV testing for female partners of consenting male partners of index clients. All partners who tested positive for HIV were encouraged to link to care at a comprehensive care clinic. Female index clients and partners with HIV (newly diagnosed or those aware of their positive HIV status) were followed up at 6 weeks, 6 months, and 12 months after enrolment by telephone or in-person, to assess linkage to care, ART initiation, and adverse events (ie, intimate partner violence or relationship dissolution). Viral load testing was conducted at 12 months follow-up (viral suppression was defined as suppression of plasma HIV RNA to <400 copies per μL). HIV risk behaviours were collected at enrolment (appendix p 3). Participants were not reimbursed for their participation in APS. We assessed effectiveness outcomes, which consisted of the proportion of male partners newly diagnosed with HIV and the proportion males partners testing for HIV for the first time among all male partners tested, and linkage to care and ART initiation at 6 weeks, 6 months, and 12 months post-enrolment among female index clients and male partners. We also report adverse events—ie, intimate partner violence and relationship dissolution. Additionally, we evaluated APS implementation outcomes of feasibility, acceptability, and integration (assessed via in-depth interviews [IDIs]) and costs (assessed via a micro-costing), and most results have been reported elsewhere.^20-22^ The current study focuses on APS effectiveness outcomes.

### Statistical analysis

We summarised continuous variables using medians and IQRs, and categorical variables using percentages. We did univariate and multivariate log binomial generalised estimated equations models with an exchangeable correlation structure and robust standard errors to examine the associations between female and facility characteristics with the identification of male partners with newly diagnosed HIV. Female self-reported risk behaviours in the past 12 months were combined into a single variable (any vs none). A priori confounders and variables statistically significant in univariate analyses (p<0·05) were considered for inclusion in the multivariate analysis. We also calculated ratios of the number of female index clients needed to interview to identify one male partner diagnosed with HIV, and one male partner with newly diagnosed HIV. Analyses were prespecified and we conducted complete case assessment, excluding missing data. Analyses were conducted using Stata BE version 17.^23^

### Role of the funding source

The funder of the study had no role in study design, data collection, data analysis, data interpretation, or writing of the report.

## Results

Between May 1, 2018, and March 31, 2020, across 31 facilities, 32 722 female participants received HIV testing and 1910 (6%) tested positive for HIV, of whom 1724 (90%) received APS. The median age of female index clients was 28 years (IQR 23–33); the majority (1317 [76%]) had a primary school education or lower. Most (1455 [84%]) were employed (table 1). The majority (1017 [59%]) of female participants were married to one partner (married monogamous); 118 (7%) were in polygamous marriages and 315 (18%) were single or had never been married. Female participants generally had low household incomes; 1402 (81%) reported 10 000 Kenyan shillings (KSh) or less a month, and few (27 [2%]) reported a history of intimate partner violence in their lifetime, and the median number of sexual partners in the past 3 years was three (IQR 2–4). The majority (1395 [81%]) of female index clients selected provider referral (having providers contacting male partners directly) as their preferred method of partner notification.

5137 male partners were elicited from female index clients, of whom 4422 (86%) received HIV testing through APS. The median age of male partners (36 years [IQR 30–41]) was higher than that of females. Most (3368 [76%]) male partners were married to one partner; 252 (6%) were in polygamous marriages and 574 (13%) were single or had never been married. The majority (2557 [58%]) of partners had a primary school education or lower, and 4008 (91%) were employed. The majority (2640 [60%]) of male partners reported a household income of 10 000 KSh or less per month. Male partners reported a median of two sexual partners (IQR 1–3) in the past 3 years.

858 (50%) female index clients and 2540 (57%) male partners reported no sexual risk behaviours; the most common risk behaviour among both groups was inconsistent or no condom use (table 1). 1439 (84%) female index clients and 3896 (88%) male partners reported having tested for HIV previously. Most (2906 [66%]) male partners received testing through APS in the community; these tests were linked to a facility and, for those who tested positive for HIV, verification of test results was completed at the facility before enrolment into care. Very few participants reported transactional sex (seven [<1%] female index clients and seven [<1%] male partners) or injection drug use (one [<1%] female index client and four [<1%] male partners; table 1).

**Figure 1:**
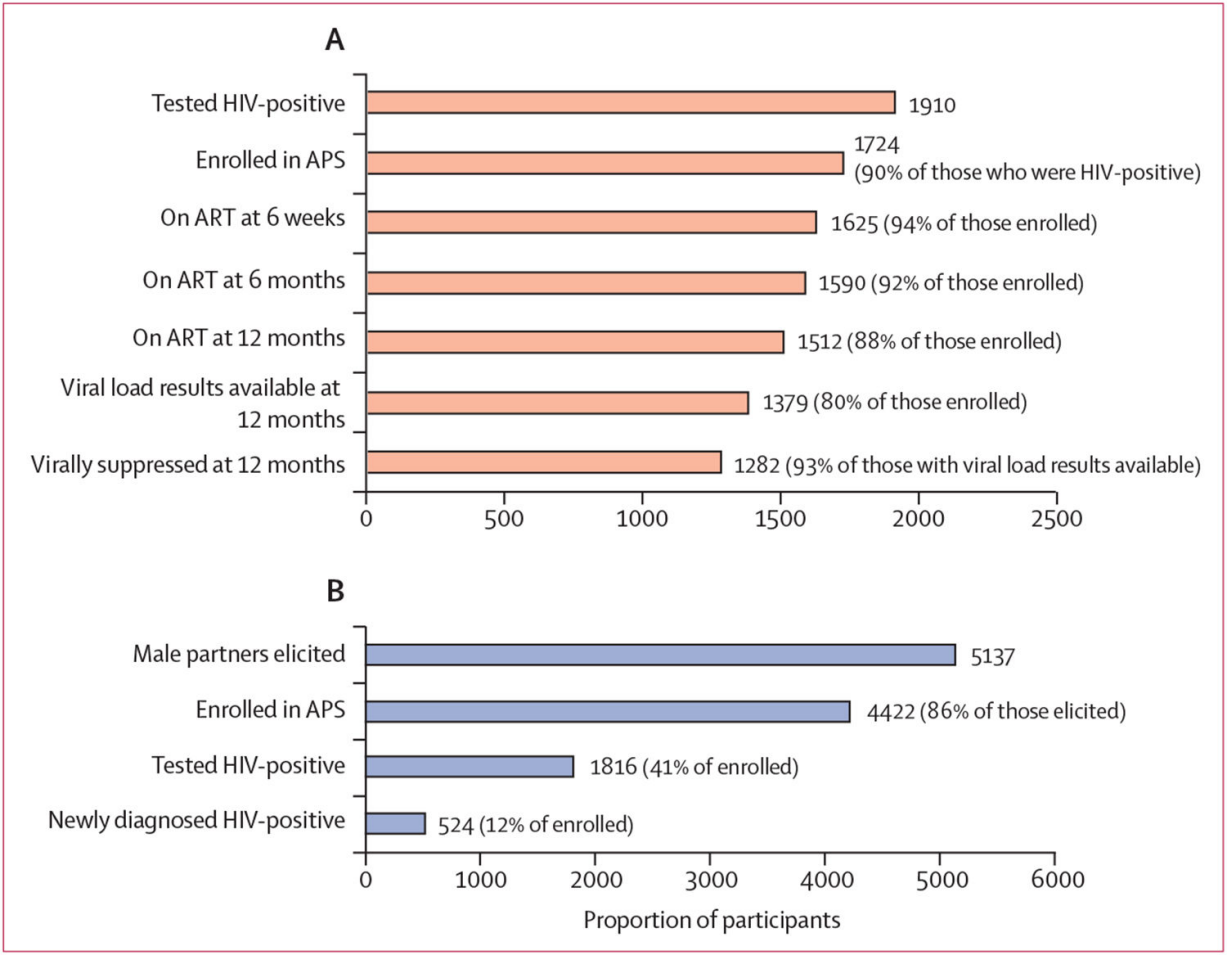
APS care cascade of female index clients and testing outcomes of male partners (A) APS care cascade and linkage to care outcomes for female index clients. (B) Male partner testing outcomes. APS=assisted partner services. ART=antiretroviral therapy.

**Figure 2:**
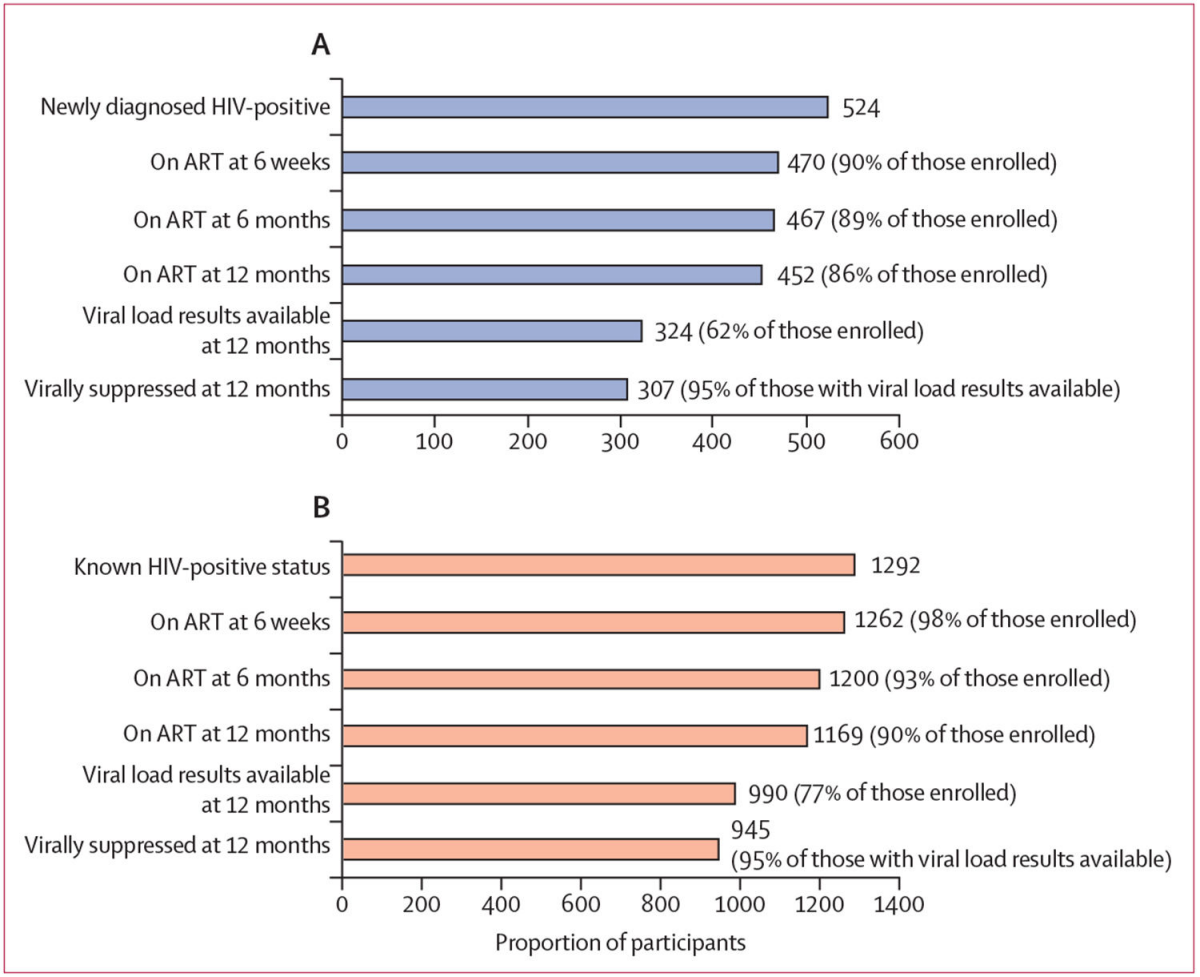
APS care cascade of male partners (A) APS care cascade and linkage to care outcomes for male partners with newly diagnosed with HIV. (B) APS care cascade and linkage to care outcomes for male partners with a previous HIV diagnosis (known HIV-positive status). APS=assisted partner services. ART=antiretroviral therapy.

APS uptake was high among female index clients (1724 [90%]) and male partners (4422 [86%]; table 2, figure 1A, B). APS identified new HIV-positive results among 524 (12%) enrolled male partners, with another 1292 (29%) male partners reporting a previous HIV diagnosis (of whom 1212 [94%] self-reported they were receiving treatment with ART at baseline). At 6 weeks follow-up, ART linkage was high among female index clients (1625 [94%]), male partners with newly diagnosed HIV (470 [90%]), and male partners with a previous HIV diagnosis (1267 [98%]; figure 2A, B). Although the proportion linked to ART declined over time, most individuals (1512 [88%] females index clients, 452 [86%] male partners with newly diagnosed HIV, and 1169 [90%] of male partners with known HIV-positive status) were taking ART at 12 months after APS. Of individuals taking ART, 12-month viral load data were not available for 345 (20%) female index clients, 200 (38%) male partners with newly diagnosed HIV, and 302 (23%) male partners known HIV-positive status. With the conservative assumption that those receiving ART without viral load data had no viral suppression, an estimated 1282 (85%) female index clients, 307 (68%) male partners with newly diagnosed HIV, and 945 (81%) male partners with known HIV-positive status on ART had viral suppression at 12 months. However, using the denominator of those among those with viral load results available, 93% of female index clients and 95% of all male partners had viral suppression. Reported intimate partner violence was low at 12 months follow-up (25 [2%] female index clients and seven [<1%] male partners), as was relationship dissolution (60 [3%] female index clients and ten [<1%] male partners).

3·3 female index clients were interviewed to identify one male partner with newly diagnosed HIV (appendix p 1). Contact information needed to be elicited for 9·8 male partners to identify one male partner with newly diagnosed HIV.

In multivariate regressions, female index clients who reported at least two sexual partners in the past 3 years (for two to five sexual partners: adjusted relative risk 1·65 [95% CI 1·33–2·04], p<0·0001; for more than five sexual partners: 1·90 [1·32–2·73], p<0·0001) were significantly more likely to lead to the identification of a male partner with newly diagnosed HIV (appendix p 1). No other characteristics related to female index clients or facilities were significantly associated with identifying a male partner with newly diagnosed HIV.

Most of our implementation outcomes have been previously published.^20,21^ Briefly, we found APS to be acceptable among female index clients and male partners who received the intervention. In IDIs, female index clients stated that they were particularly motivated by desire to preserve the health of their partners via exposure notification, HIV testing, and linkage to ART if needed. Some female index clients mentioned that the anonymity of provider notification was a benefit of APS, especially for those who feared repercussions from disclosing HIV exposure. Both male partners and female index clients valued the counselling associated with APS, which made them feel comfortable to share their sexual history and reduced their fears of confidentiality breaches to partners (unpublished data). Similarly, IDIs among providers found APS to be acceptable.^20^ Providers mentioned being motivated by the health benefits of APS in identifying and linking people with HIV to care. They stated that APS is an efficient method of targeted testing to people exposed to HIV who otherwise might not test for HIV at a clinic. However, providers noted that APS was time consuming, requiring lengthy counselling, sometimes over multiple sessions to build rapport, as well as long-distance travel to locate partners in the community. Other challenges included dealing with the sensitive nature of partner elicitation, locating partners with incomplete contact information, and dealing with hostility and even violence from partners. We also conducted a micro-costing to assess APS implementation costs in 14 clinics from the payer perspective.^21^ Average programme costs were US$31·59 per male partner tested, with personnel accounting for the majority of costs (47%), followed by transport (13%). This finding is consistent with the intensive counselling and partner tracing time described in provider IDIs.

## Discussion

We assessed the real-world performance of APS when integrated into routine clinical care in western Kenya. We found that APS is a safe, effective, and high-yield strategy to reach male partners who have been potentially exposed to HIV with testing and linkage to care. The high APS uptake observed in our programme among female index clients (90%) and male partners (86%) were similar to those of APS demonstration projects conducted in Cameroon,^11^ Malawi,^7^ and Mozambique.^24^ We also found high ART retention by 12 months follow-up, which demonstrates that APS provision in real-world settings can effectively link male partners with HIV to treatment.

Viral suppression at 12 months follow-up was high among those with viral load results available. However, some individuals were missing viral load results, likely due to the emergence of the COVID-19 pandemic, which resulted in shortages of reagents and prioritising of laboratory supplies for COVID-19 testing. Therefore, viral load testing was largely halted in Kenya in April, 2020. When we conservatively assumed that those without viral load results were not suppressed, we found the lowest viral suppression was among male partners with newly diagnosed HIV, followed by male partners with a known HIV-positive status, with female index clients having the highest viral suppression. Our findings are consistent with studies showing lower viral suppression among male individuals compared with female individuals,^3^ with the highest loss to follow-up in the first year of ART. However, newly diagnosed male partners also had the highest proportion of missing viral load data (with over a third missing), so they are most impacted by the assumption that individuals without viral load data are not virally suppressed. Utilising the denominator of those with viral load results available, over 90% of all three groups were virally suppressed, which is higher than Kenya national programme data (85% virally suppressed among those with available viral load results.^25^ A study evaluating national programme data from Kenya showed that male individuals were half as likely to get a viral test compared with female individuals.^26^ The incomplete assessment of viral load adds uncertainty to our results; however, male individuals with newly diagnosed HIV also have slightly lower confirmed retention on ART at 12 months (86%) compared with male individuals with known HIV-positive status (90%) and female individuals (88%) at 12 months, and retention in care declined in all groups over time. This finding suggests that additional support might be necessary to encourage sustained retention in care.

In our study, the new HIV-positive result among enrolled male partners (12%) is slightly lower than some of the reported literature. An APS programme in Cameroon found that 20% of all partners had new HIV-positive results,^11^ and a study in Malawi found 50% of individuals had new HIV-positive results, although only 35% of all elicited partners were tested.^7^ In their APS randomised controlled trial in central and western Kenya in 2015,^9^ Cherutich and colleagues reported 23% of partners had new HIV-positive results. Our findings might differ in part because knowledge of HIV status has increased over time; in 2012, 47% of people with HIV were aware of their status in Kenya^27^ compared with 80% in 2018.^28^ Additionally, male individuals generally have a lower prevalence of HIV compared with female individuals, which might affect the reported rate of new HIV-positive results. However, the 12% of new HIV-positive results via APS in our study is substantially higher than what would be expected through facility HIV testing. The high rate of new HIV-positive results found in APS programmes probably results from the targeted testing of sexual partners who might be either exposed to HIV or the source of the index client’s HIV infection. As knowledge of HIV status increases, APS will probably become an even more efficient testing strategy in sub-Saharan Africa. Indeed, APS is implemented in many high-income countries with low rates of undiagnosed HIV prevalence. Interestingly, we found a lower number of index clients needed to interview to identify a newly positive male partner compared with Cherutich and colleagues (3·3 *vs* 5·1, respectively).^9^ The result might be because we elicited more partners per index, (3 *vs* 1·7 in the study by Cherutich and colleagues^9^). The number of index clients needed to interview is dependent on the prevalence of undiagnosed HIV and the sexual network in the region. Further, we found that the majority of male partners had previously tested for HIV, in contrast with Cherutich and colleagues’ study,^9^ which reported high rates of first-time testers. This result is probably reflective of increasing HIV testing coverage among the population. Finally, a substantial proportion of male partners had known about their HIV-positive status and were already receiving ART; therefore, we did not find a beneficial effect of retesting individuals with known HIV to encourage linkage to care.

Additionally, we found that most (66%) male partners were tested in the community as opposed to a facility. This finding highlights an important benefit of APS in that it provides the option of community-based testing. Previous research shows that male individuals face barriers in accessing facility-based testing, including time and transport costs.^29,30^ Targeted community-based testing of individuals who have been exposed to HIV might overcome these barriers without the substantial costs of widespread community-based testing. We also report that female index clients’ age, marital status, reported risk behaviours, or geographical region were not associated with the identification of a male partner with newly diagnosed HIV, which indicates that APS should probably be universally offered to female individuals who test HIV-positive.

Similar to previous trials and demonstration projects,^9,11,13,24,31^ we report low rates of intimate partner violence and relationship dissolution among people receiving APS. Consistent with other studies, we excluded individuals at high risk of intimate partner violence, so we are unable to evaluate APS outcomes in this population. APS provision has been shown to be safe and effective among female individuals with a history of intimate partner violence and is associated with a low risk of social harms.^32,33^ However, research has also shown that intimate partner violence is a barrier to APS uptake, both due to female individuals refusing APS because of fear of intimate partner violence, or health-care worker assessments that female individuals were at moderate or high risk of intimate partner violence and therefore ineligible for APS. Previous research from our team found that 10% of APS non-enrolment among female individuals was due to intimate partner violence.^34^ The proportion of female individuals reporting a lifetime history of intimate partner violence in our study is low (2%), partly because females at moderate or high risk of intimate partner violence were excluded from APS; however, this outcome might also be due to social desirability bias. Continued training of health-care providers on offering counselling on sensitive topics, such as intimate partner violence screening, is needed to further assess the potential impact of intimate partner violence on APS uptake.

This study has several strengths and limitations. First, we used programmatic data, so the quality of the data in this study were not as robust as that of data in a clinical trial. However, we conducted weekly data quality checks and data cleaning to improve accuracy of the collected data. Further, we collected data on long-term outcomes, including ART uptake, viral suppression, and adverse events. Second, with regard to generalisability, we only collected data on female index clients and their male partners, so we cannot assess the performance of APS among male index clients or female partners. Additionally, although APS is offered to pregnant female individuals programmatically, our study excluded pregnant female individuals; thus, we cannot evaluate APS’s impact in this population. The vast majority of participants reported heterosexual relationships, with low representation from key populations (female sex workers, people who inject drugs, and fisher folk), which limits generalisability to these groups. Additionally, we report low proportions of self-reported HIV risk behaviours despite the presence of an HIV diagnosis, which might be due to social desirability bias. However, we include a large sample size across many clinics of varying types in two counties in Kenya; our findings among female index clients and male partners are probably generalisable to other similar settings.

A significant strength is that the study was designed and implemented in partnership with the government (Ministry of Health of Kenya), a non-governmental organisation (PATH), and public health researchers (University of Washington, Seattle, WA, USA). APS was conducted by government-employed health-care workers who also provided general HIV care services at the facilities, enabling an assessment of APS scale-up outside of trial settings. Additionally, APS providers were trained on APS and were certified providers of HIV testing and counselling. Further, participants were not reimbursed for their participation, so uptake and retention probably reflects real-world conditions. However, although we report results from a real-world implementation study, our implementation team invested a substantial amount of funds in capacity building and staff training and resources for partner tracing. We held several ongoing trainings to ensure staff developed strong skills for discussing sensitive topics, including partner elicitation, intimate partner violence screening, and partner notification and encouragement for HIV testing. These trainings increased APS uptake among female index clients and male partners. Our results likely represent the upper bound of those achievable in large-scale programmatic implementation of APS.

Guidelines for HIV testing in Kenya recommend voluntary APS implementation as part of routine HIV testing services, but APS is a resource-intensive intervention, requiring counsellors to conduct community-based tracing of partners and provision of HIV testing. In settings with a shortage of health-care workers and a restricted budget, APS implementation might present challenges.^35^ Our micro-costing of APS implementation showed that average facility costs increase when integrating APS into HIV clinics, largely driven by personnel costs, followed by transport.^21^ Future research is needed to understand the impact of these costs and resources used on sustaining APS scale-up and quality over time.

In summary, we find real-world integrated APS to be a safe and effective method to identify male partners with HIV and achieve high retention in care. As knowledge of HIV status increases in sub-Saharan Africa, targeted strategies like APS are necessary to identify people at high risk of HIV. Our results can inform programmatic decisions regarding APS scale-up in Kenya and similar settings.

## Supplementary Material

1

## Figures and Tables

**Table 1:** Demographics, sexual behaviour, and HIV testing outcomes of female index clients and male partners receiving APS from 31 health facilities in western Kenya

	Female index clients (n=1724)	Male partners (n=4422)
Median age, years (IQR)	28 (23–33)	36 (30–41)
Marital status		
Single or never married	315 (18%)	574 (13%)
Married monogamous	1017 (59%)	3368 (76%)
Married polygamous	118 (7%)	252 (6%)
Cohabitating	10 (1%)	14 (<1%)
Divorced or separated	134 (8%)	160 (4%)
Widowed	130 (8%)	54 (1%)
Highest education completed		
Did not complete primary school	491 (29%)	763 (17%)
Completed primary school	826 (48%)	1794 (41%)
Completed secondary school	291 (17%)	1295 (29%)
Post-secondary school	116 (7%)	570 (13%)
Occupation		
Formally employed	725 (42%)	1707 (39%)
Self-employed	730 (42%)	2301 (52%)
Unemployed	156 (9%)	319 (7%)
Student	113 (7%)	95 (2%)
Monthly household income		
0 KSh to ≤10 000 KSh	1402 (81%)	2640 (60%)
>10 000 KSh to ≤50 000 KSh	312 (18%)	1714 (39%)
>50 000 KSh to 100 000 KSh	10 (1%)	68 (2%)
Key populations		
Sex workers	5 (<1%)	0
Fisher folk*	5 (<1%)	45 (1%)
History of intimate partner violence		
Yes	27 (2%)	0
No	1697 (98%)	4422 (100%)
County		
Homa Bay	774 (45%)	2584 (58%)
Kisumu	950 (55%)	1838 (42%)
HIV-associated risk behaviours in the past 12 months		
Inconsistent condom use	355 (21%)	621 (14%)
No condom used during last sexual encounter	252 (15%)	566 (13%)
History of pre-exposure prophylaxis use	1 (0·1%)	27 (1%)
Injection-drug-use needle sharing	1 (<1%)	4 (<1%)
Sexually transmitted infection	13 (1%)	27 (1%)
Recurrent use of post-exposure prophylaxis use	2 (<1%)	6 (<1%)
Sex under the influence of drugs	9 (1%)	17 (<1%)
Multiple sexual partners	109 (6%)	326 (7%)
Sexual partner with HIV	39 (2%)	249 (6%)
Transactional sex	7 (<1%)	7 (<1%)
None	858 (50%)	2540 (57%)
Median number of sexual partners in the past 3 years (IQR)	3 (2–4)	2 (1–3)
Previously tested for HIV	1439 (84%)	3896 (88%)
Result of last HIV test		
Do not know	9 (1%)	12 (<1%)
Negative	1421 (82%)	2637 (60%)
Positive	9 (1%)	1247 (28%)
Not applicable	285 (17%)	526 (12%)
HIV self-test in the past 12 months	80 (5%)	418 (10%)
Tested as a couple during APS†	89 (5%)	103 (2%)
HIV testing location for APS		
Non-facility based‡	352 (20%)	2906 (66%)
Facility based	1372 (80%)	1516 (34%)
Type of APS selected (female index clients only)		
Provider referral	1395 (81%)	..
Client referral	126 (7%)	..
Dual referral	88 (5%)	..
Couple testing	64 (4%)	..
Contract referral	49 (3%)	..

Data are n (%) or median (IQR). APS=assisted partner services.

* Defined as people who catch or sell fish for a living.

† Female index clients who attended the clinic with male partners could receive couples testing. Male partners could receive couples testing in the community or the clinic if their female partners selected dual referral or couples testing as their preferred APS type.

‡ All HIV tests done in the community were linked to a facility, and verification of the test results was completed at the facility’s comprehensive care clinic before enrolment to care and treatment.

**Table 2:** APS care cascade and outcomes among female index clients and male partners

	Female index clients	Male partners*
Baseline		
Tested for HIV	32 722	4422
Tested HIV-positive	1910 (6%) of 32 722 who tested for HIV	1816 (41%) of 4422 who enrolled
Enrolled in APS	1724 (90%) of 1910 who tested HIV-positive	4422 (86%) of 5137 elicited
Male partners with a previous HIV diagnosis	..	1292 (29%) of 4422 who enrolled
Male partners receiving ART at APS enrolment	..	1212 (94%) of 1292 with known HIV-positive status
Male partners newly diagnosed HIV-positive	..	524 (12%) of 4422 who enrolled
**6-week outcomes**		
Attended 6-week follow-up	1704 (99%) of 1724 who enrolled	1790 (99%) of 1816 who were HIV-positive
Linked to HIV care†		
Total	1632 (95%) of 1724 who enrolled	1744 (96%) of 1816 who were HIV-positive
Known HIV-positive	..	1267 (98%) of 1292
Newly diagnosed HIV-positive	..	477 (91%) of 524
On ART		
Total	1625 (94%) of 1724 who enrolled	1732 (95%) of 1816 who were HIV-positive
Known HIV-positive	..	1262 (98%) of 1292
Newly diagnosed HIV-positive	..	470 (90%) of 524
Relationship ended since APS enrolment	21 (1%) of 1704 who attended follow-up	2 (<1%) of 1790 who attended follow-up
Experienced intimate partner violence since APS enrolment	5 (<1%) of 1704 who attended follow-up	4 (<1%) of 1790 who attended follow-up
**6-month outcomes**		
Attended 6-month follow-up	1641 (95%) of 1724 who enrolled	1717 (95%) of 1816 who were HIV-positive
On ART		
Total	1590 (92%) of 1724 who enrolled	1667 (92%) of 1816 who were HIV-positive
Known HIV-positive	..	1200 (93%) of 1292
Newly diagnosed HIV-positive	..	467 (89%) of 524
Relationship ended since APS enrolment	40 (2%) of 1641 who attended follow-up	4 (<1%) of 1667 who attended follow-up
Experienced intimate partner violence since APS enrolment	10 (<1%) of 1641 who attended follow-up	5 (<1%) of 1667 who attended follow-up
**12-month outcomes**		
Attended 12-month follow-up	1535 (89%) of 1724 who enrolled	1636 (90%) of 1816 who were HIV-positive
On ART		
Total	1512 (88%) of 1724 who enrolled	1621 (90%) of 1816 who were HIV-positive
Known HIV-positive	..	1169 (90%) of 1292
Newly diagnosed HIV-positive	..	452 (86%) of 524
Relationship ended since APS enrolment	60 (3%) of 1535 who attended follow-up	10 (<1%) of 1636 who attended follow-up
Experienced intimate partner violence since APS enrolment	25 (2%) of 1535 who attended follow-up	7 (<1%) of 1636 who attended follow-up
Viral load results available		
Total	1379 (80%) of 1724 enrolled	1314 (72%) of 1816 who were HIV-positive
Known HIV-positive	..	990 (77%) of 1292
Newly diagnosed HIV-positive	..	324 (62%) of 524
Virally suppressed among those with viral load results available		
Total	1282 (93%) of 1379	1252 (95%) of 1314 who were HIV-positive
Known HIV-positive	..	945 (95%) of 990
Newly diagnosed HIV-positive	..	307 (95%) of 324
Virally suppressed among those on ART		
Total	1282 (85%) of 1512	1252 (77%) of 1621 who were HIV-positive
Known HIV-positive	..	945 (81%) of 1169
Newly diagnosed HIV-positive	..	307 (68%) of 452
At least one of my partners received APS since my enrolment	..	1157 (64%) of 1816 who were HIV-positive

APS=assisted partner services. ART=antiretroviral therapy.

* 5137 (mean of three partners per female index client) male partners were elicited.

† Linkage to care is defined as enrolled in a comprehensive care clinic for HIV care.

## Data Availability

The study protocol, statistical plan, and deidentified data from the APS Scale up Project are available upon request by contacting the corresponding author.

## Research in context

### Evidence before this study

We searched PubMed for studies published in English from Jan 1, 2000, to Jan 1, 2022, using the terms “contact tracing”, “partner notification”, “partner services”, “HIV”, “trials”, “implementation”, “integration”, “Africa”, and “scale-up”. Assisted partner services (APS) demonstration projects conducted in Zimbabwe, Cameroon, Tanzania, and Mozambique show that APS provision within health systems is a safe and effective strategy to reach individuals exposed to HIV with testing and care. We found no data on long-term outcomes, including antiretroviral therapy (ART) retention and viral suppression among those receiving APS.

### Added value of this study

In this large-scale implementation study using government health-care workers to provide APS across 31 facilities, we reported high APS uptake among female index clients and male partners. At 12 months post-APS provision, the majority of female index clients (88%) and male partners (89%) with HIV were taking ART, with few reported adverse events. Viral suppression was high among those with viral load results available.

### Implications of all the available evidence

APS provision through national health systems can be an effective and safe method to reach partners who have been potentially exposed to HIV with notification and testing. We report high retention on ART at 12 months and viral suppression rates that are similar to national programme data in Kenya. These results can inform programmatic decisions regarding APS scale-up in sub-Saharan Africa.
