# Supplementary material for: Real-world impact of integrating HIV assisted partner services into 31 facilities in Kenya: a single-arm, hybrid type 2 implementation-effectiveness study
Source: Lancet Glob Health. Author manuscript; Available in PMC 2023 May 3. (PMC10156000; doi:10.1016/S2214-109X(23)00153-5)
Supplement: 1 [file NIHMS1892579-supplement-1.pdf]

THE LANCET
Global Health

Supplementary appendix

This appendix formed part of the original submission and has been peer reviewed. We post it as supplied by the authors.

Supplement to: Sharma M, Naughton B, Lagat H, et al. Real-world impact of integrating HIV assisted partner services into 31 facilities in Kenya: a single-arm, hybrid type 2 implementation-effectiveness study. *Lancet Glob Health* 2023; **11:** e749–58.

SUPPLEMENTAL APPENDIX

Accompanying the manuscript

Real-world impact of integrating HIV Assisted Partner Services into 31 facilities in Kenya: a single-arm, hybrid type 2 implementation-effectiveness study

Supplemental Table 1. Number Needed to Interview (NNTI) ratios

	NNTI Ratio
**Overall female index client APS engagement**	
Female index client (N=1724) per male HIV-positive partner (N=1816)	0·9
Female index client (N=1724) t per newly diagnosed male HIV-positive partner (N=524)	3·3
**Overall male partners identified per new HIV-positive diagnosis**	
Elicited male partners (N=5137) per new HIV-positive diagnosis (N=524)	9·8
Enrolled male partners (N=4422) per new HIV-positive diagnosis (N=524)	8·3

Supplemental Table 2. Associations between female and facility characteristics and identifying a newly positive male partner

		Univariate model			Multivariate model		
	Proportion	Relative Risk	95% Confidence Interval	P value	Adjusted Relative Risk	95% Confidence Interval	P value
**Female index client characteristics**							
Age (years)[a]							
<20	10·1%		Ref			Ref	
20-29	49·6%	0·95	(0·71,1·29)	0·752	0·94	(0·68,1·128)	0·679
30-39	28·1%	0·93	(0·64,1·35)	0·710	0·90	(0·59,1·36)	0·613
40+	12·2%	0·85	(0·58,1·22)	0·374	0·83	(0·58,1·19)	0·315
Education[a]							
Did not complete primary	28·5%		Ref			Ref	
Completed primary	47·9%	0·91	(0·76,1·10)	0·327	0·87	(0·74,1·03)	0·114
Completed secondary	16·9%	0·97	(0·77,1·22)	0·774	0·90	(0·72,1·13)	0·368
Post-secondary	6·7%	1·06	(0·78,1·44)	0·727	0·97	(0·72,1·30)	0·840
Monthly household income[a]							
0 to ≤10,000 KSh	81·3%		Ref			Ref	
>10,000 to 50,000 KSh	18·1%	1·14	(0·94,1·39)	0·191	1·15	(0·96,1·38)	0·124
>50,000 to 100,000 KSh	0·6%	1·66	(1·00,2·75)	0·051	1·57	(0·95,2·62)	0·081
Marital status							
Single/never married	18·3%		Ref				
Married monogamous/cohabitating	59·6%	1·14	(0·89,1·45)	0·300			
Married polygamous	6·8%	1·14	(0·87,1·49)	0·360			
Divorced/separated/widowed	15·3%	0·95	(0·67,1·34)	0·780			
Risk behaviors							
Risk behaviors	49·8%	0·90	(0·76,1·07)	0·216			
No risk behaviors	50·2%		Ref				
Number of sexual partners[b]							
<2	20·4%		Ref			Ref	
2 to 4	61·7%	1·67	(1·36,2·06)	0·000	1·65	(1·33,2·04)	0·000
5+	17·9%	1·89	(1·32,2·71)	0·001	1·90	(1·32,2·73)	0·001
History of IPV							
Yes	1·6%	1·16	(0·73,1·86)	0·531			
No	98·4%		Ref				
Employment							
Self employed	42·3%	0·99	(0·83,1·67)	0·872			
Unemployed	9·1%	0·87	(0·63,1·20)	0·383			
Student	6·6%	1·05	(0·63,1·76)	0·856			
Employed	41·1%		Ref				
**Facility characteristics**							
County							
Kisumu	55·1%	0·85	(0·85,0·98)	0·158			
Homa Bay	44·9%		Ref				
Location							
Rural	65·2%	0·88	(0·68,1·15)	0·364			
Urban	34·8%		Ref				

[a] *Variables defined* a priori *for inclusion in adjusted model*
[b] *Significant (P<0.05) variable included in adjusted model*

APS implementation context: Guidelines and targets

The Kenya Ministry of Health introduced Guidelines for APS in May 2018. The guidelines did not include APS targets but rather focused on capacity building. In October 2019, PEPFAR introduced targets for APS which indicated that 20% of individuals testing HIV-positive should come from APS; this was increased to 35% in 2020. In 2021, PEPFAR targets were removed after it was decided that target setting may create pressure on healthcare providers to recruit and test individuals through APS potentially interfering with the goal of maintain APS as a voluntary and discrete program.

Kenya guidelines for HIV testing and counseling services (HTS)

HIV testing is Kenya is offered by two approaches: Client initiated HTS, in which individuals seek out HIV testing at a clinic or in the community and provider-initiated HTS, in which a healthcare provider offers HIV testing to clients in a facility and provides an "opt-out" option.

HTS is performed according to the national testing algorithm. Individuals undergo an HIV screening test (Determine) and provided results in the same visit. Those who test HIV-negative are given counseling to assess their understanding of results. Those testing HIV-positive are provided with a confirmatory test (First response); individuals who test positive on the confirmatory test are provided counseling and linked to treatment. In cases where the confirmatory results disagree with the screening results, clients are referred to a laboratory for further testing and potential repeating of the testing algorithm.

APS implementing team:

- Study team: University of Washington, PATH, Kenya Ministry of Health. The team had expertise in HIV, policy and programming, implementation science, epidemiology, biostatistics, and economic evaluation.
- National officers and head of the National AIDS and STI Control Program (NASCOP), HTS manager, HIV prevention lead
- National HTS committee of experts/technical working groups
- County officers: County AIDS and STI Coordinators in Kisumu and Homabay
- Sub-county officers: Sub-County AIDS and STI Coordinators
- Health workers: HTS providers, lay counselors and nurse counselors, clinicians, community health workers

Other organizations involved in APS implementation:

- Implementing partners include: The Elizabeth Glaser Pediatric AIDS Foundation in Kenya, LVCT Health-Kenya, Jaramogi Oginga Odinga Teaching & Referral Hospital (JOOTRH), and AIDS Healthcare Foundation-Kenya
- Communities: Community advisory boards, Community activists, persons living with HIV

<u>APS enrollment survey questions:</u>

Intimate Partner Violence Assessment

Have you ever been in a relationship with a person who has physically hurt you?

If yes, when did this occur?

Have you been in a relationship with a person who threatens, frightens, or insults you, or treats you badly?

If yes, when did this occur?

Have you been in a relationship with a person who forces you to participate in sexual activities that make you feel uncomfortable?

If yes, when did this occur?

Do you think any of these things could happen to you if you decide to receive assisted partner notification services?

Risk Behaviors in the 12 months prior to testing (Select all that apply)

Sex partner(s) is HIV-positive

Sex partner(s) at high risk for HIV and HIV status currently unknown

Have sex with more than one partner

Transactional sex (exchanged sex for money)

Recent STI

Recurrent use of PEP

Ever used PrEP*

Recurrent sex under influence of alcohol/recreational drugs

Inconsistent or no condom use

Did not use condom during last sex

IDU with shared needles/syringes

None of the above

**Use of PrEP was categorized as an HIV risk behavior as it is indicative of condomless sex.*
